# Effect of proprioceptive neuromuscular facilitation technique on the treatment of frozen shoulder: a pilot randomized controlled trial

**DOI:** 10.1186/s12891-022-05327-4

**Published:** 2022-04-20

**Authors:** Ping Lin, Moudan Yang, Deqing Huang, Huan Lin, Jialin Wang, Chaoping Zhong, Li Guan

**Affiliations:** 1 Department of Rehabilitation Medicine, The Fushun People’s Hospital, 47 Xiangyun Street West section, Zigong, China; 2 Department of Radiology, The Fushun People’s Hospital, 490 Jixiang Road, Fushi Street Zigong, Fushun, China

**Keywords:** Frozen shoulder, Magnetic resonance imaging, Proprioceptive neuromuscular facilitation, Rehabilitation

## Abstract

**Background and objectives:**

Frozen shoulder is a common painful disease of the shoulder joint characterized by structural changes in the shoulder joint, restricting both active and passive shoulder joint activities. Proprioceptive neuromuscular facilitation (PNF) effectively improved and maintained the range of motion; however, it is not clear whether it can improve the shoulder joint structure in patients with frozen shoulder. This pilot study used magnetic resonance imaging (MRI) observation to assess the improvement of the local structure of the shoulder joint upon PNF treatment to elucidate a target based on structure for the treatment of frozen shoulder.

**Materials and methods:**

Forty-eight patients with frozen shoulder were randomly divided into the traditional manual therapy group and the PNF technique group. Changes in the thicknesses of the coracohumeral ligament (CHL) and capsule in axillary recess (CAR) of the shoulder joint were observed via MRI upon admission and at 4 weeks after treatment. A visual analog scale (VAS) and passive shoulder range of motion (ROM) at abduction, anteflexion and external rotation position were used to evaluate the improvement of shoulder joint pain and function in the initial, mid-term, and discharge of the two groups of patients.

**Results:**

The primary outcome results shown that the PNF joint mobilization significantly reduced the thickness of the CHL (*p* = 0.0217) and CAR (*p* = 0.0133). Compared with simple joint mobilization, The mid-term and discharge rehabilitation assessment results showed that PNF has a better effect on shoulder pain. At the mid-term evaluation, the ROM of the PNF group was significantly better than that of the Control group in the three directions (*p* < 0.05).

**Conclusion:**

As an adjunctive therapy, PNF can improve the shoulder joint structure of patients with frozen shoulder and is an effective treatment strategy for frozen shoulder.

## Introduction

Frozen shoulder, also known as adhesive capsulitis of the shoulder, is a condition with an uncertain etiology and is characterized by painful, gradual loss of both active and passive glenohumeral motion [1, 2]. The prevalence of frozen shoulder in the general population is about 2-3% [3]. Most patients with this condition are elderly females, seriously affecting their working ability and quality of life [4]. At present, the pathogenetic mechanism of frozen shoulder is not yet clear, which is why it is often labeled as idiopathic or primary frozen shoulder [5]. The main pathologic findings of frozen shoulder are thickening of glenohumeral joint capsule, contracture and adhesion to humeral head rather than periarthritis inflammation [6]. Although the current diagnosis of frozen shoulder is mainly based on the appearance of clinical symptoms, Magnetic resonance imaging (MRI) is widely used to study the structure of the shoulders of patients who have this condition [7–9]. The coracohumeral ligament (CHL) starts from the lateral edge of the root of the coracoid process, projects obliquely outwards and downwards, and reaches the ligament in the front of the greater tuberosity of the humerus. It was observed through MRI that the CHL of patients with frozen shoulder was significantly thickened, and it was closely associated with the limited mobility of the shoulder joint [8]. Capsule in axillary recess (CAR) thickening of the capsule wall also significantly aggravates the discomfort brought about by the symptoms of frozen shoulder [10]. Therefore, improving the abnormal structural changes of the shoulder joint is important for treating frozen shoulder.

Frozen shoulder is currently considered to be a self-limited disease since it can spontaneously resolve without any intervention [11]. However, most patients who have this condition seek medical treatment because of its long recovery period and discomforting symptoms. The treatment options currently performed for frozen shoulder include physical therapy, intraarticular corticosteroid injection (the injections comprise hydrodilation with normal saline, hyaluronic injection, also the injection site could be rotator interval), capsulotomy closed mobilization, and rehabilitation manual therapy [6]. Nonsurgical therapies are the mainstay treatments for frozen shoulder. Surgical management, such as arthroscopic capsular release or capsular incision, is considered only after failure of nonsurgical treatment, and the surgical procedures are heterogeneous, with inconsistent benefits and iatrogenic risks [12]. Manual therapy is increasingly used to facilitate the quicker recovery of patients, despite a lack of solid evidence to support its effectiveness [13, 14]. Proprioceptive neuromuscular facilitation (PNF) is an important technology used in rehabilitation manual therapy [15]. It is a stretching technique that can improve muscle elasticity and has been shown to positively affect the active and passive range of motion in patients with frozen shoulder [16–18]. Rehabilitation therapists have used PNF to restore the functional activity range of patients with soft tissue injuries and increase their overall strength, balance, and coordinate muscle strength [13]. However, current research on the use of PNF to treat frozen shoulder is only focused on improving symptoms, and the specific mechanisms involved are still being elucidated. To date, there is still no report regarding the impact of PNF technique on the structure of the shoulder joint in patients with frozen shoulder.

We designed this pilot randomized controlled trial to evaluate the effect of PNF on the treatment of frozen shoulder via MRI examination and an assessment of the improvement of the abnormal shoulder joint structure. We hypothesize that PNF technique can better alleviate the symptoms of frozen shoulder and be a tool to improve shoulder joint structure. This study also aims to provide a theoretical basis for frozen shoulder therapies and the promotion of PNF technique in the future.

## Materials and methods

### Research design

This study was a single-center, pilot randomized controlled trial. The clinical trial protocol complied with the CONSORT clinical trial guidelines [19] and was approved by the Ethics Committee of Fushun People’s Hospital (FS2019012) in December 2019. The trial protocol was registered at the Chinese Clinical Trial Registry (www.chictr.org.cn, 24/12/2020, ChiCTR2000041369). All participants signed a statement of informed consent before participating in the study.

### Patients

Patients admitted to the Department of Rehabilitation Medicine diagnosed with frozen shoulders according to the guidelines presented in a previous study [20] were deemed eligible for inclusion in the study. The inclusion criteria were 40-65 years old, with shoulder joint pain and limited mobility for more than 4 weeks (stiffness stage of frozen shoulder). The exclusion criteria were: the presence of cardiovascular and cerebrovascular and other serious systemic diseases; severe mental illness or impaired consciousness; shoulder tumors, tuberculosis or rheumatic disease, neck disease or other diseases radiated to the shoulder; shoulder trauma that has not been cured, severe osteoporosis, or bone lesions as observed from X-rays of the shoulder joint bone; participate in other clinical trials; those who have received other treatments within 2 weeks before the start of their participation in the study. Patients who received an intraarticular steroid injection within 6 weeks would also be excluded; and those whose complete data cannot be obtained. Patients who failed to complete the treatment after inclusion, those who requested to discontinue, or those who withdrew voluntarily were also excluded from the study.

### Procedures

The blinding method in this study involved a non-disclosure of the implementation of the treatment plan to the subjects and data collectors not participating in the grouping and treatment of subjects. Independent rehabilitation therapists carried out the data analyses. Eligible patients were assigned to one of the two groups using the assignment code contained in a consecutively numbered sealed opaque envelope. Simple randomization was then performed using a computer. Patients were randomly divided into the Control group and the PNF group. The demographic characteristics of the two groups of patients were first recorded. A rehabilitation therapist will provide manual therapy to patients in the Control group. In the PNF group, under the concept of the international classification of functioning (ICF), an overall assessment of the patient’s degree of limitation of shoulder joint movement and the degree of specific limitation of movement, the strengths and weaknesses of the patients, and the expected level of patient functional recovery was performed. The assessment content is carried out from five aspects: body structure and function level, activity level, participation level, personal factors and environmental factors. Based on the Evaluation, PNF technique was used to carry out functional recovery training for the problems determined. The treatment cycle of the two groups of patients was 4 weeks, during which they were assisted with extracorporeal shock wave and ultrasound physical therapy once daily. All treatment operations were performed by professional rehabilitation therapists with more than 2 years of work experience.

### Interventions

#### Manual therapy

**Fig. 1:**
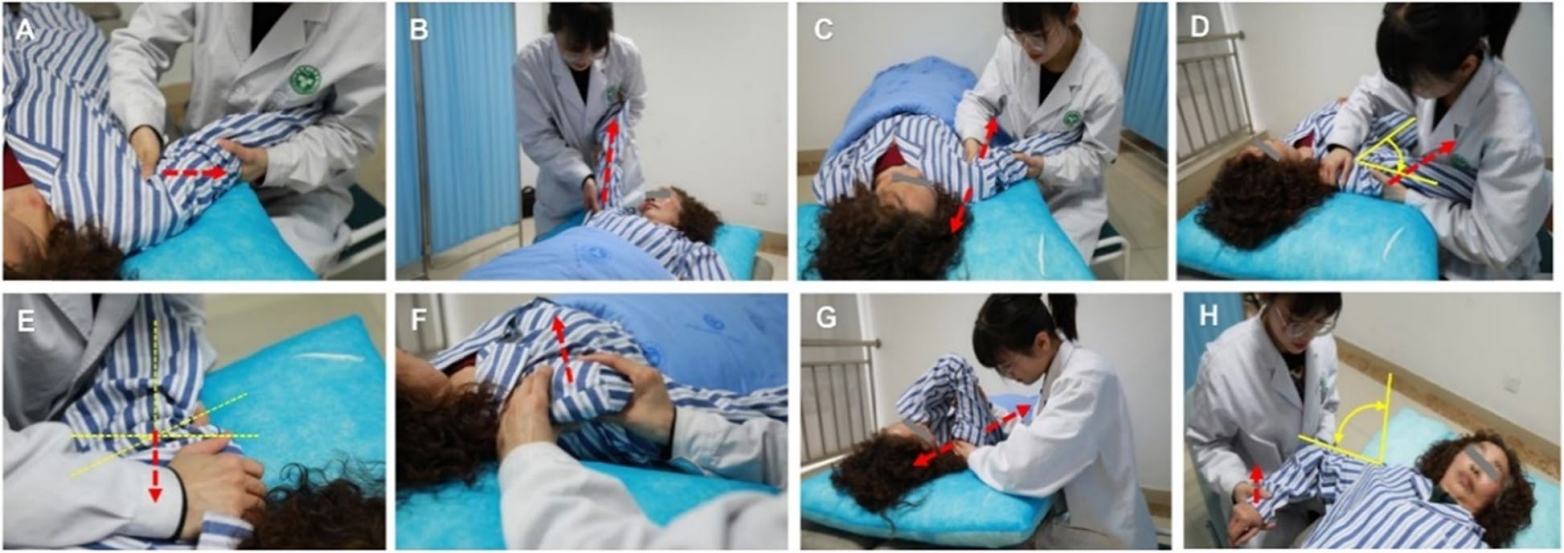
**A-H** Traditional manual therapy, The direction of the rehabilitation therapist’s auxiliary activity (red arrow), angle activity (yellow arrow)

Manual therapy included separation traction, long axis traction, up-and-down sliding, abduction sliding to the side of the foot, front-to-back sliding, back-to-front sliding, side sliding, and internal rotation swings **(**Fig. 1**)**. The Maitland four-level technique was implemented according to the patient’s shoulder joint condition. The joint movement limitation caused by pain was treated using grade I and grade II techniques, joint pain and stiffness were treated using grade III techniques, and the joint movement limitation caused by the surrounding soft tissue adhesion and contracture was treated using grade IV techniques.

#### PNF technique under the ICF concept

**Fig. 2:**
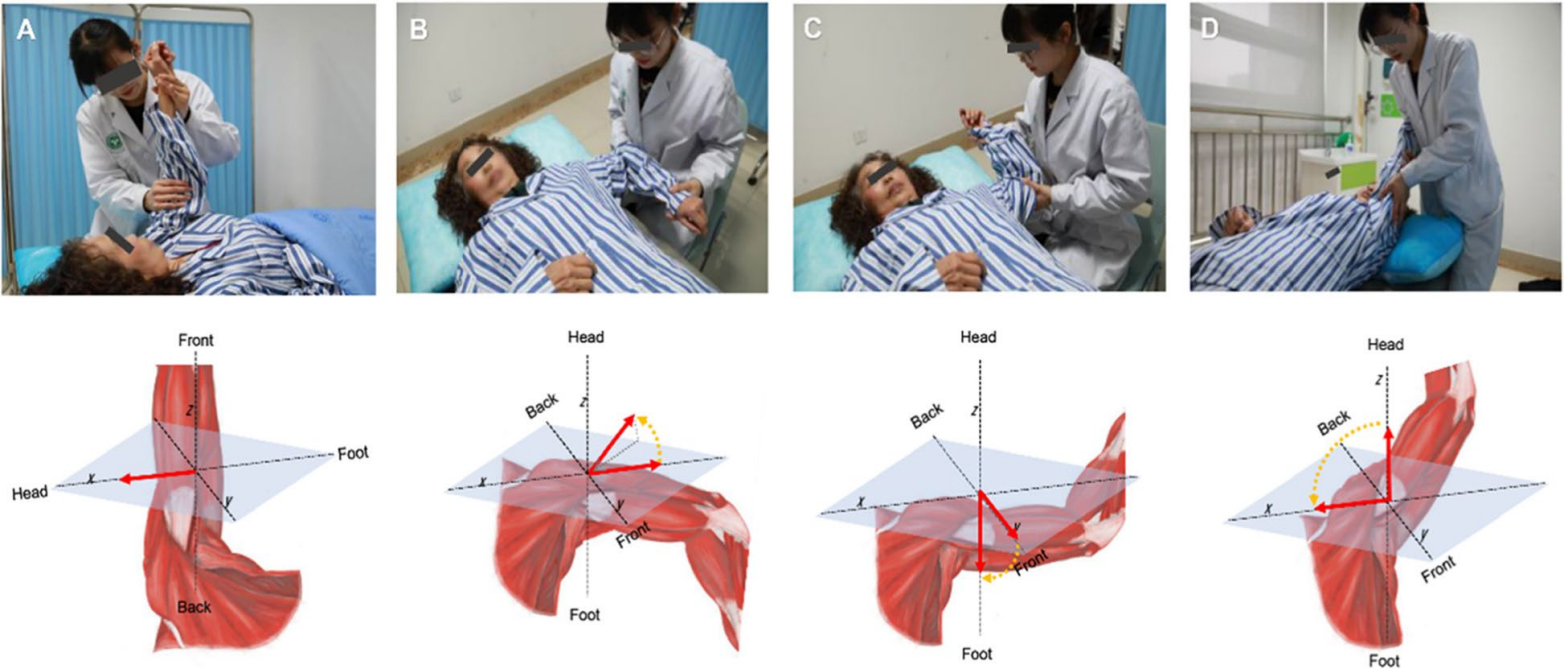
**A-D** PNF technique under ICF concept fully mobilizes restricted muscles to participate in rehabilitation training

The use of PNF technique under the ICF concept combines dynamic reversal, stability reversal, rhythmic stability, hold-relaxation, contraction-relaxation, and other techniques when performing joint mobilization operations on patients. PNF technique involves stably reversing the exercise, giving continuous resistance, fully mobilizing the restricted muscles, and requiring the patient to relax and quickly giving the restricted side a pull, and maintaining it **(**Fig. 2**)**.

Compared with traditional manual therapy, PNF technique can better stimulate the body’s proprioceptors, promote the relevant neuromuscular responses, and enhance the contraction ability of the corresponding muscles. Meanwhile, by adjusting the abnormal excitability of the sensory nerves, the muscle tension can be changed, and the muscle spasm can be relieved. Make it move in a normal way. It is expected to be more helpful in relieving pain in patients with frozen shoulder and improving joint ROM.

**Fig. 3:**
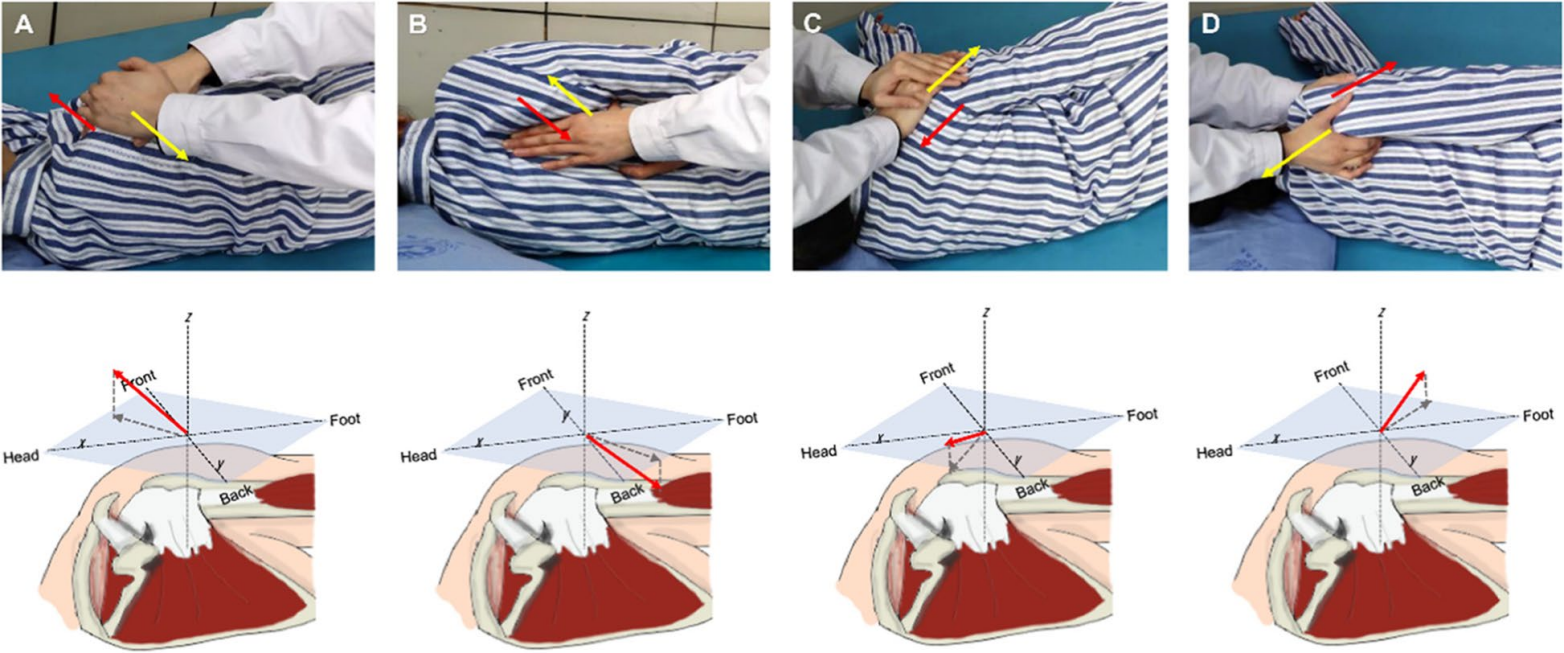
**A-D** Extension and contraction of the shoulder girdle. The direction of the external force applied by the rehabilitation therapist (yellow arrow), and the direction of the patient’s shoulder against the external force (red arrow)

The rehabilitation training method adopts a diagonal movement of the upper limbs, the extension and contraction of the shoulder girdle, and the extension and flexion of the upper limbs. The scapular girdle patterns performed during the training include forward extension, retraction, forward retraction, and forward extension, encouraging patients to mobilize the shoulder joint against in the direction of the therapist’s operation **(**Fig. 3**)**.

**Fig. 4:**
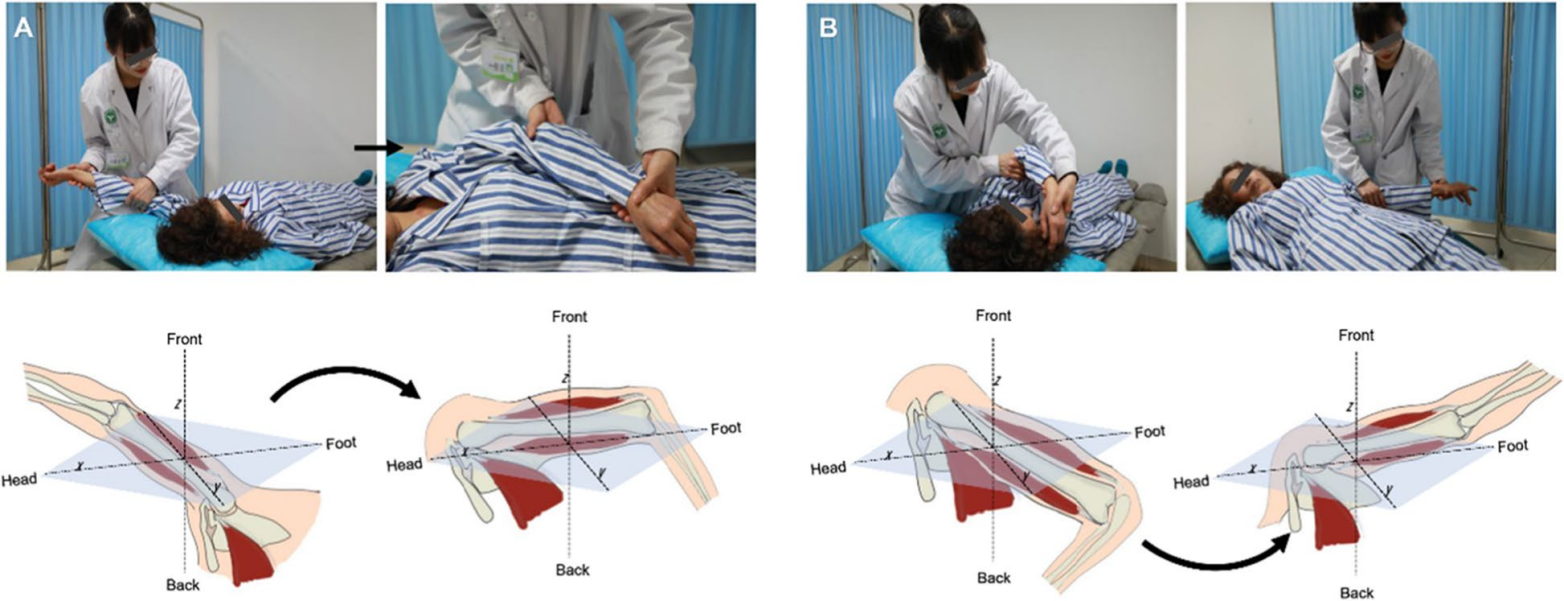
**A-B** Extension and flexion patterns of the upper limbs. The upper limbs move according to the diagonal angle

The upper limb flexion and extension exercises include flexion-abduction-external rotation, extension-adduction-internal rotation, flexion-adduction-external rotation, and extension-abduction-internal rotation. A four-level loosening technique was simultaneously performed in the range of flexion-abduction-external rotation to extension-adduction-internal rotation and flexion-adduction-external rotation to extension-abduction-internal rotation once a day, with each loosening cycle lasting for approximately 30 min **(**Fig. 4**)**.

### Outcome measurements

#### Primary outcome measures: structural changes in the shoulder joint

Upon patient admission to the hospital and after 4 weeks of treatment, a professional radiologist who did not know the group status performed an MRI examination of the affected shoulder joint. All patients’ arm positions were standardized, with thumbs pointing upwards. According to the method described in the study by Mengiardi [10], T1-weighted images in the sagittal oblique plane (600–700/12, 3–4 mm section thickness, 160×160 mm field of view) and T1-weighted spin-echo images in the coronal oblique plane (777–800/12–20, 3–4 mm section thickness, 160×160 mm field of view) were obtained.

Two radiologists with 5 years of MRI result analysis experience analyzed the MRI images obtained. They did not know the patients’ diagnoses and did not participate in the treatment process. The two radiologists measured the thickness of CHL and CAR in parallel. When the measurement difference was large, another radiologist with 10 years of experience performed a re-measurement and correction. The thickness change of the structure was the thickness value after treatment subtracted from the baseline value upon patient admission.

#### Second outcome measures: pain assessment of the shoulder joint

Pain is a major symptom of frozen shoulder. In addition to the pain experienced in daily life, pain during treatment was more pronounced. To further clarify the effect of the PNF technique on the improvement of frozen shoulder symptoms compared with traditional manual treatment, we used a VAS scoring method. This method was used to evaluate the two groups of patients at the initial rehabilitation assessment (at admission), during a mid-term assessment (2 weeks after treatment), and at the discharge assessment (at 4 weeks after treatment). The average night rest pain assessment using a VAS involves a line segment with ten scales, with two ends being “0” and “10,” respectively. A score of “0” means no pain, and a score of “10” means the most severe, unbearable pain. Data collection was conducted by data assessors who did not know the groups and did not participate in the treatment of the patients.

#### Other outcome measures: evaluation the shoulder range of motion (ROM)

Limitation of active and passive movement was a common symptom in patients with frozen shoulder. In this study, patients were included at the stiffness stage, and the difference in active activity may not be obvious. Therefore, we only evaluated the passive ROM of the two groups of patients. Passive ROM was measured in the standing position with the use of a goniometer. Abduction, anteflexion and external rotation were recorded respectively in the ROM of the shoulder joint. External rotation was measured in the horizontal plane, with the elbow at the side. Abduction was measured in the frontal plane and anteflexion in the sagittal plane.

#### Sample size calculation

Limited by the current lack of MRI evaluation data related to the application of PNF in the treatment of frozen shoulder, our sample size calculation was based on VAS score. The previous pre-experimental results showed that the VAS score of the Control group was 2.5 ± 1.2 and that of the PNF group was 1.6 ± 0.9 after treatment. The G-Power software was used to compare the independent samples of the two groups, α = 0.05 and 1-β = 0.2, and the loss to follow-up ratio was expected to be 5%. Finally, the minimum sample size of each group was calculated to be 22 patients.

### Statistical analysis

GraphPad-Prism (version 9.1, USA) software was used for the statistical analysis of data. Data were expressed as the means and the standard deviations. The Kolmogorov-Smirnov test was used to test whether the data conformed to a normal distribution. The Fish-LSD test was performed on the demographic binary data, and the unpaired Student’s *t* test was used to compare two groups of continuous variables. Non-normally distributed data are presented as medians with interquartile ranges (IQR) and differences between the groups were assessed by use of Mann Whitney ***U*** tests. A *P-* valve< 0.05 was set to be statistically significant.

## Results

### Baseline conditions of the included patients

**Fig. 5:**
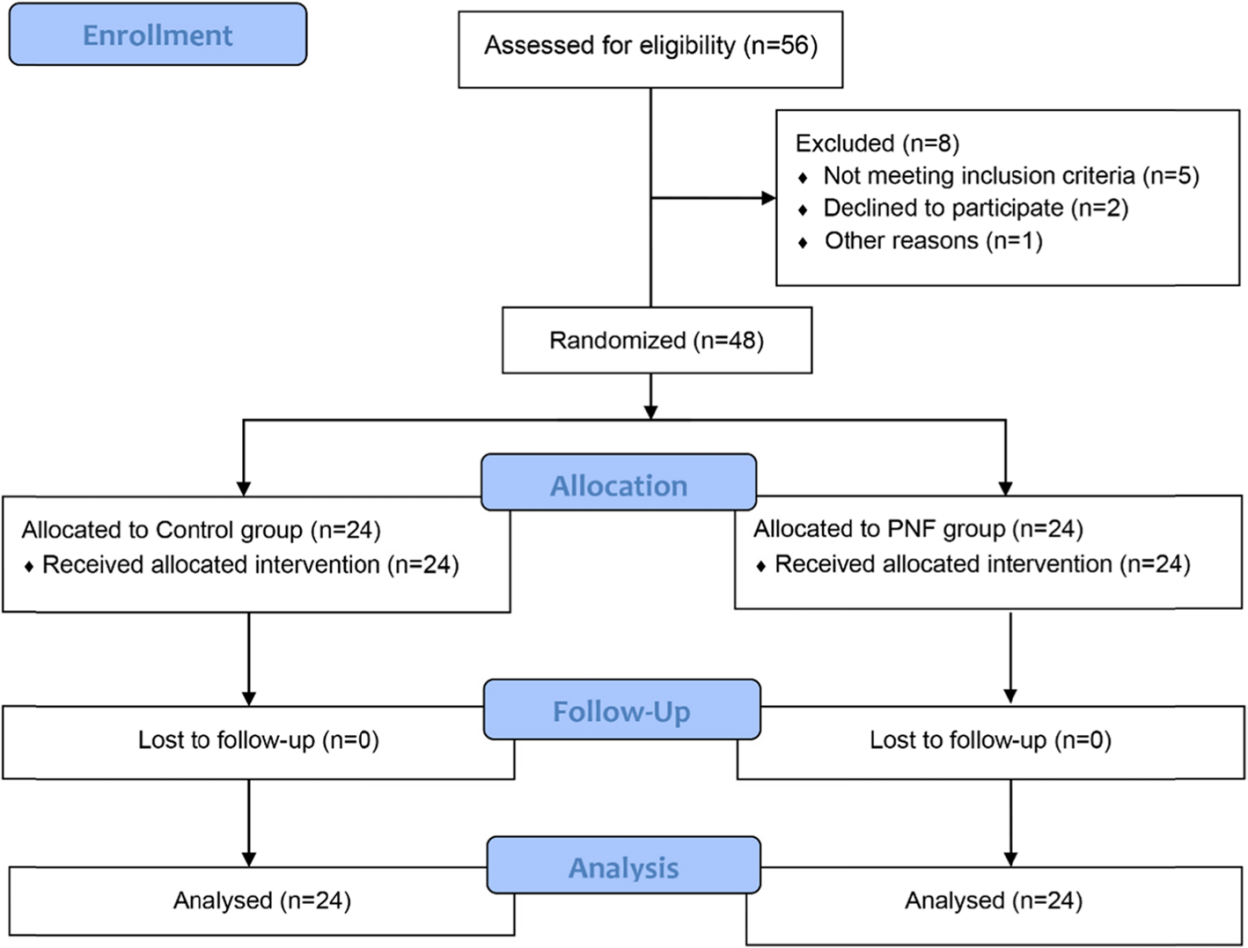
CONSORT flow diagram

The study was eventually discontinued due to funding depletion. Due to limited funds, we continuously enrolled 24 patients in a row and stopped MRI evaluation after receiving two MRI examinations (12 patients in each group), but other outcomes continued to be included until the study was terminated due to the depletion of funds. A total of 56 patients with frozen shoulder were recruited in this study. Five did not meet the inclusion criteria, two withdrew halfway through the study, and one patient was transferred to the orthopedic practitioners and received surgery. Finally, 48 patients were included in the randomization and the grouping. In each of the Control and PNF groups, twelve patients of each group received shoulder MRI examinations. All patients in both groups participated until the end of the study **(**Fig. 5**)**.

The baseline demographic characteristics of the two groups of patients were the same. There were no significant differences in age (*p* = 0.260), gender composition ratio (*p* = 0.546), height (*p* = 0.754), weight (*p* = 0.451), and duration of the disease (*p* = 0.591) in the Control group and PNF groups. The detailed demographic characteristics were listed in **(**Table 1**)**.

**Table 1:** Demographic characteristics

Characteristics	Control group (*n* = 24)	PNF group (*n* = 24)	*p*-Valve
Age (years)	54.7 ± 6.5	52.3 ± 5.2	0.260
Sex (M/F)	11/13	10/14	0.546
Height (cm)	159.8 ± 5.6	160.6 ± 6.4	0.754
Weight (kg)	57.4 ± 6.0	56.5 ± 6.1	0.451
Duration (day)	45.2 ± 13.8	46.1 ± 11.1	0.591

*PNF* Proprioceptive neuromuscular facilitation, *M* Male, *F* Female

### Primary outcome: the changes of the abnormal structure of shoulder joint

**Fig. 6:**
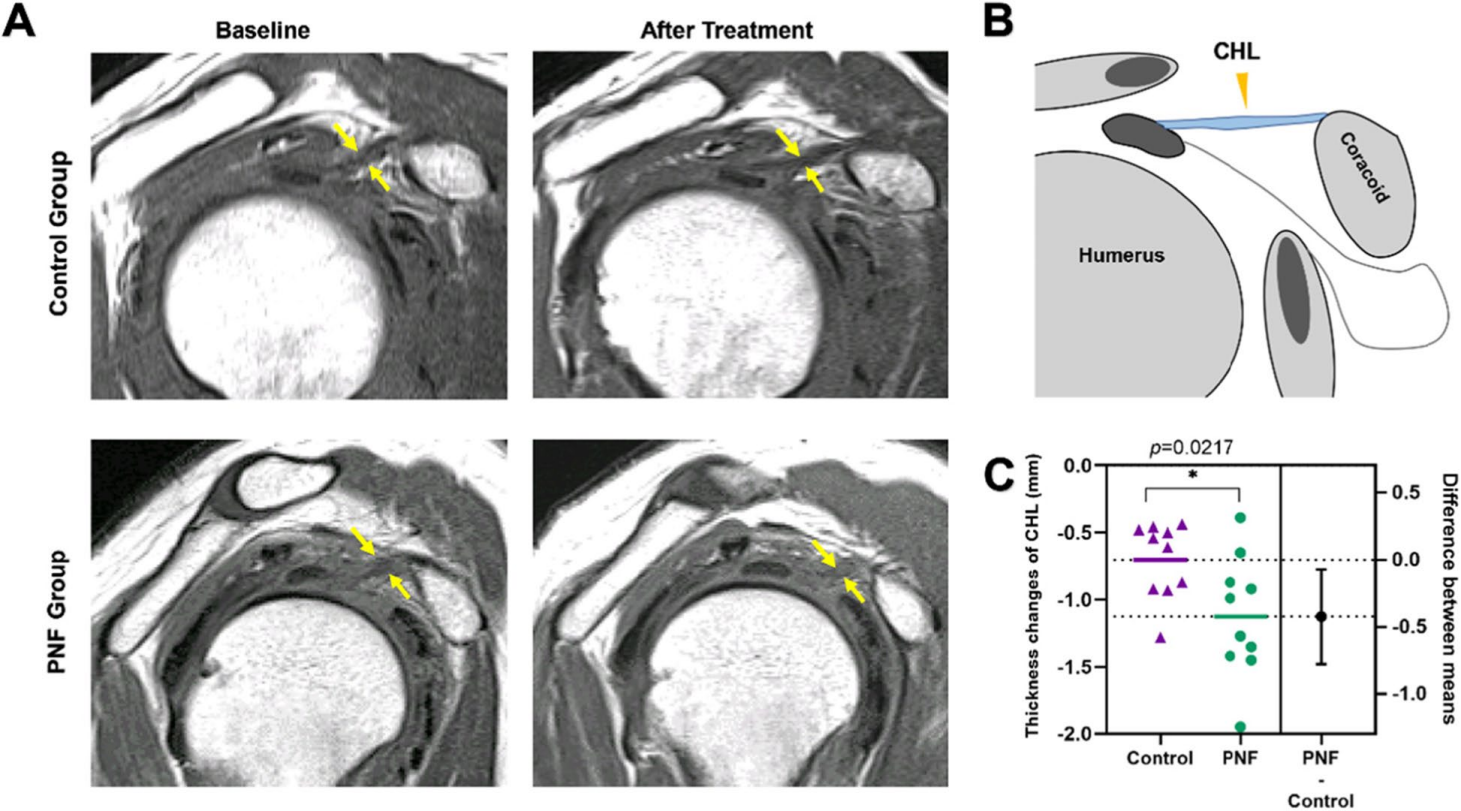
CHL thickness changes before and after treatment in the two groups. **A** Typical MRI images of CHL before and after treatment in the two groups, Sagittal oblique T1-weighted image shows thickened CHL (yellow arrows); **B** Sagittal oblique view shows the positional relationship of CHL; **C** CHL thickness changes in the two groups of patients, the data is represented by Mean ± SD, and ^*^p < 0.05 indicates comparison with the Control group

**Fig. 7:**
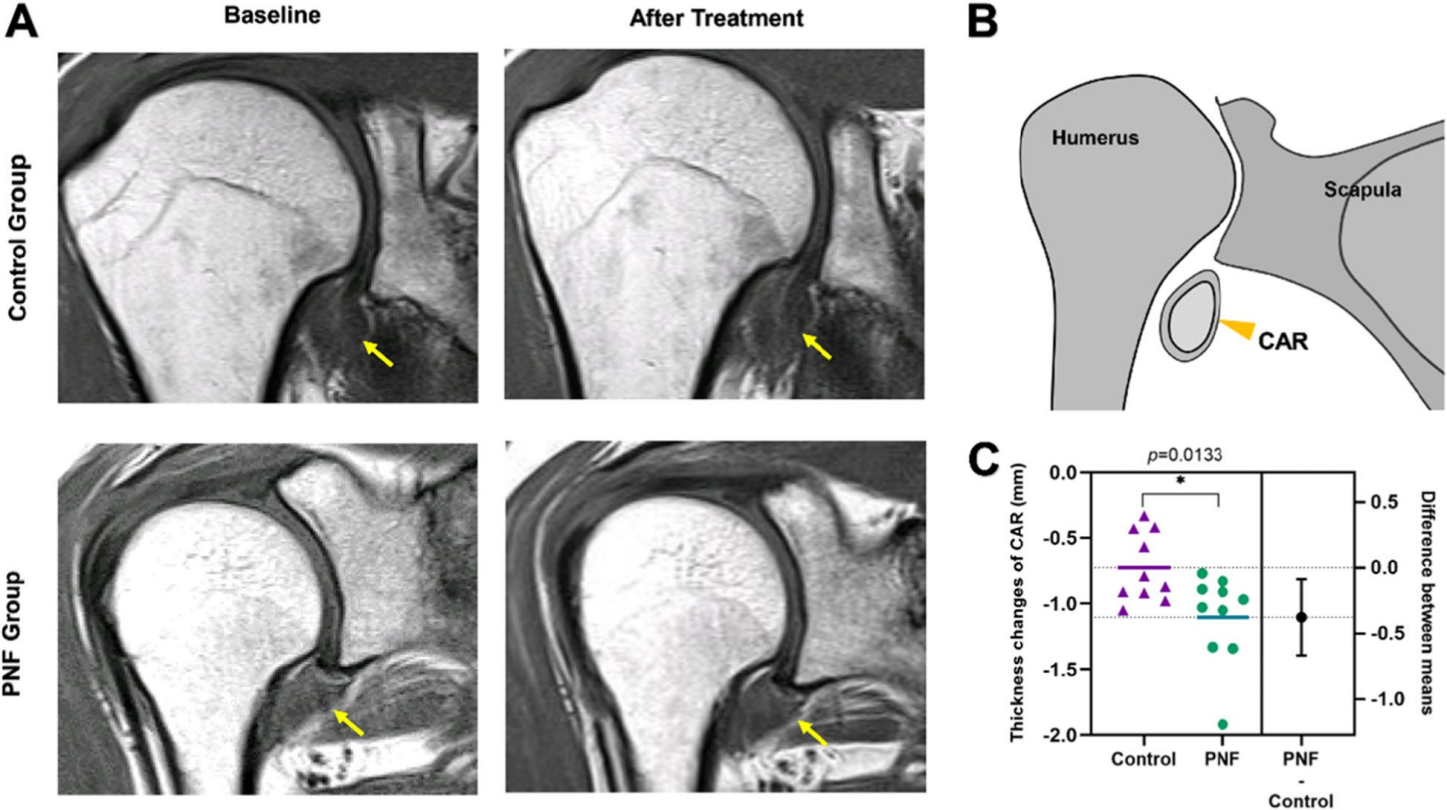
CAR thickness changes before and after treatment in the two groups. **A** Typical MRI images of CAR before and after treatment in the two groups, T1-weightedspin-echo images in the coronal oblique shows thickened CAR (yellow arrows); **B** Coronal oblique shows the positional relationship of CAR; **C** CAR thickness changes in the two groups of patients, the data is represented by Mean ± SD, and ^*^p < 0.05 indicates comparison with the Control group

The improvement of pain and ROM in frozen shoulder patients with PNF may be related to the improvement of the abnormal structure of shoulder joint. We compared the changes in the shoulder joint structure of the two groups of patients using MRI before and after treatment. The primary measurement indicators are the changes in the thicknesses of the CHL and CAR calculated as the thickness after 4 weeks of treatment minus the thickness at baseline. The results showed that the PNF treatment significantly reduced the thickness of the CHL compared with that of traditional manual therapy (*p* = 0.0217, mean of diff. = − 0.42, 95% CI of diff. Between mean = − 0.78 to − 0.07). This may be the structural basis for improving the ROM **(**Fig. 6**)**. The change in CAR thickness in the PNF group was significantly better than that in the Control group (*p* = 0.013, mean of diff. = − 0.38, 95% CI of diff. Between mean = − 0.67 to − 0.09) **(**Fig. 7**)**.

### Second outcome: VAS of control group and PNF group

According to the pain assessment results, there was no significant baseline difference in shoulder pain between the two groups (*p* = 0.564, mean of diff. = − 0.16, 95% CI of diff. Between groups = − 0.41 to 0.74). The VAS scores of the patients in the PNF group during the mid-term assessment (*p* = 0.019, mean of diff. = − 0.67, 95%CI of diff. Between group = − 1.22 to − 0.11) and discharge assessment (*p* = 0.007, mean of diff. = − 0.88, 95% CI of diff. Between groups = − 1.50 to − 0.24) were significantly better than those in the Control group. The specific VAS score comparison can be seen in (Table 2**)**.

**Table 2:** VAS scores of shoulder joint in Control group and PNF group

Time point	Control group (*n* = 24)	PNF group (*n* = 24)	*p*-Valve	Difference between group (95% CI)
Baseline	6.95 ± 1.04	7.12 ± 0.94	0.564	−0.16 (− 0.41 to 0.74)
Mid-term	4.95 ± 0.99	4.29 ± 0.90	0.019^*^	−0.67 (−1.22 to − 0.11)
Discharged	2.37 ± 1.20	1.50 ± 0.93	0.007^*^	−0.88 (−1.50 to − 0.24)

Data expressed as Mean ± SD; * Statistically significant

### Other outcome: shoulder ROM of control group and PNF group

We observed the ROM of patients in both groups, and PNF was significantly better than Control group at mid-term evaluation (*p*<0.05). In the final evaluation, all shoulder ROM measurements were still in favor of the PNF group, but were not significant(*p*>0.05) **(**Table 3**)**.

**Table 3:** Passive ROM of shoulder joint in Control group and PNF group

Position	Time point	Control group (*n* = 24)	PNF group (*n* = 24)	*p*-Valve
Abduction	Baseline	50 (39-60)	50 (41-101)	0.41
	Mid-term	70 (43-90)	100 (78-140)	0.01^*^
	Discharged	85 (80-149)	130 (85-170)	0.32
Anteflexion	Baseline	70 (70-80)	94 (45-120)	0.24
	Mid-term	90 (76-112)	140 (102-165)	0.02^*^
	Discharged	100 (88-158)	155 (111-168)	0.16
External	Baseline	0 (0-5)	8 (0-20)	0.14
	Mid-term	13 (5-28)	40 (31-42)	0.01^*^
	Discharged	18 (8-32)	40 (25-66)	0.04^*^

Data expressed as Medians (with interquartile range); * Statistically significant

## Discussion

To date, the exact pathogenesis of frozen shoulder is still unclear. Histological and immunocytochemical studies have demonstrated that active fibroblast proliferation was accompanied by the conversion of fibroblasts to myofibroblasts [21]. It has also been confirmed that there is inflammation and fibrosis in the shoulder joints of patients with frozen shoulder [22]. The structural changes of the soft tissues in the shoulder joint are the primary cause of the limited mobility and pain experienced by patients with frozen shoulder. Improving the ROM of the shoulder joint and reducing pain have long been the focus of attention in treating this condition. A recent meta-analysis discussed the treatment methods for frozen shoulder, presenting current methods such as physical therapy, intraarticular corticosteroid injection, capsulotomy closed mobilization, and rehabilitation manual therapy [6]. Although the intra-articular injection of corticosteroids is an effective treatment, complications of steroid use may require further consideration, and physical therapy may still be the first recommended treatment [23]. A better way to treat shoulder pain was to focus on loosening the adhesive joint tissue, increasing the volume of the joint cavity, and improving ROM [20]. There is still no high-quality evidence comparing the effects of joint loosening under anesthesia and rehabilitation manual therapy in improving shoulder joint tissue adhesion. Manual therapy is still the most widely used method in treating frozen shoulder, considering both treatment cost and patient acceptance [24, 25]. Our team attempted to integrate PNF technology into traditional manual therapy and explored the effect of PNF treatment on improving the abnormal tissue structure observed in frozen shoulder. As far as we know, there is still no research report on whether using MRI to observe PNF technology can improve the joint and soft tissue structure of patients with frozen shoulder.

The current research revealed that there were many changes in the joint tissue of patients with frozen shoulder that can be characterized via MRI Assessment of CHL and CAR thickening had high diagnostic specificity [7]; CHL thickening is currently an important piece of imaging evidence for the diagnosis of frozen shoulder. The thickening of the CHL and CAR has a high correlation with the limitation of joint motion [9, 26]. A meta-analysis showed that PNF technology could significantly improve the ROM of patients with frozen shoulder, but the underlying mechanism was unclear [13]. Our research results showed that PNF technology significantly improved the thickness of the CHL and CAR, which provided a convincing explanation for supporting the use of PNF for the treatment of frozen shoulder. Due to the contracture and morphological changes in the soft tissues of the shoulder joint during frozen should, patients with this condition have a reduced ability to control their shoulder movements, especially their ability to perceive diagonal movements of their shoulder joints [27]. PNF technique emphasizes diagonal movement according to the patient’s rehabilitation goals. Here, the patient controls and relaxes, enhancing their ability to recover their perception and control of the diseased area [28, 29]. PNF technique was also able to closely restore patients’ daily living ability, thus mobilizing their recovery [30].

Our research found that PNF treatment was more helpful in restoring the abnormal changes in the soft tissue structure of the shoulder joint than manual therapy alone. A reduction in the thickness of the CHL and CAR also helped restore the ROM of the shoulder joint. PNF technique introduces resistance training, where patients need to coordinate their overall structure and movement to resist the motion given. This training can mobilize the diseased tissues so that that they could be restored through gradual training [29].

Our study further explained why PNF helps restore the ROM of patients with frozen shoulder, providing a new strategy and therapeutic target for treating frozen shoulder. This study had several limitations. First, this study was a single-center pilot study with a small sample size. A clinical study involving a larger sample is needed to verify the results obtained in this study. In addition, we did not conduct further subgroup analyses on the gender, nature of work, and education level of the included patients. Finally, we were not able to objectively evaluate the patients’ subjective feelings; the comfort of patients was also an important measure to evaluate a potential treatment measure. Finally, it was worth noting that through our study, PNF was clearly beneficial for patients with frozen shoulder, but the current evidence was not enough to support PNF can be used independently for the treatment of frozen shoulder, and more clinical studies may be needed. PNF therapy might be an adjunctive therapy for patients with frozen shoulder, instead of a primary and independent treatment.

## Conclusions

This study found that PNF technique was more helpful in restoring the joint structure of patients with frozen shoulder compared to traditional manual therapy. PNF technique was also more helpful than traditional manual therapy in relieving the pain. Therefore, we conclude that PNF technique can be used as an adjunctive effective treatment for frozen shoulder.

## Data Availability

The datasets generated and/or analysed during the current study are not publicly available due the researchers’ institution’s data management center requested that the data be retained and are available from the corresponding author on reasonable request (guandoctor@126.com).
